# Heterotopic ossification following COVID-19 infections: systematic literature review of case reports and case series

**DOI:** 10.1186/s12891-024-07537-4

**Published:** 2024-05-29

**Authors:** Hachem Chaitani, Laurent Fabeck, Simon Koulischer

**Affiliations:** 1 Université Libre de Bruxelles, 808 route de Lennik, Anderlecht, 1070 Belgium; 2 Department of Orthopaedic Surgery, Saint-Pierre University Hospital, 105 rue aux Laines, Brussels, 1000 Belgium

**Keywords:** Heterotopic ossification, Ectopic ossification, Myositis ossificans, COVID-19, SARS-CoV-2, Coronavirus

## Abstract

**Background:**

This review aims to study the clinical characteristics, diagnostic results, treatments, and outcomes in patients with heterotopic ossification following COVID-19 infection.

**Methods:**

A literature search for eligible articles was conducted using MEDLINE/Pubmed, Global Health, and Scopus databases (January 12th, 2023), including all case reports and case series from any country and language. The criteria for inclusion in this review were cases of COVID-19 infection subsequently developing heterotopic ossification.

**Results:**

This systematic review analysed 15 reports (*n* = 20 patients) documenting cases of heterotopic ossification following COVID-19 infection. 80% of the patients were male, with a median age of 59 years. All patients required intensive care unit stay with an average duration of 48.5 days. Mechanical ventilation was necessary for all patients and 30% of them underwent tracheostomy. Common symptoms included stiffness and pain, most frequently affecting multiple locations (70%), with the hips and shoulders being predominantly involved. X-rays were the most commonly used imaging modality, followed by computed tomography. Although treatment was given, some of the patients continued to experience symptoms, particularly stiffness.

**Conclusion:**

20 patients who developed heterotopic ossification after COVID-19 have been reported, the majority of which had at least two independent risk factors for this condition. The link between those two clinical entities is therefore uncertain, requiring further investigation. It is nonetheless important to suspect heterotopic ossification in patients with severe COVID-19 infection, prolonged immobilisation, mechanical ventilation, who develop joint pain and stiffness, as this condition can significantly impact patients’ quality of life.

**Protocol registration:**

CRD42023393516.

## Introduction

The coronavirus disease 2019 (COVID-19) global pandemic erupted in December 2019, resulting in numerous infections caused by severe acute respiratory syndrome coronavirus two (SARS-CoV-2). Although the viral infection affected mostly the lower respiratory tract causing acute respiratory distress syndrome (ARDS), many extrapulmonary complications have been described after COVID-19 infections. They may be the result of the viral infection itself, systemic inflammation, or other factors including intensive care unit (ICU) stay and prolonged bed rest [1, 2]. The aetiology of heterotopic ossification (HO) is still not clearly understood. It can be defined as the emergence of bone tissue in ectopic tissue such as muscles. It occurs most commonly following traumatic brain or spinal cord injuries, intense trauma, severe thermal injuries, surgeries (e.g. hip arthroplasty), and immobilisation [3, 4].

No link has been described yet between COVID-19 infection and HO, as the two phenomena remain unclear generally. We conducted a systematic review of case reports to summarise the evidence in the literature of the association between severe COVID-19 infections and HO development. Although a systematic review cannot demonstrate a causal relationship between these two processes, it can help make a few hypotheses requiring further research investigations. One potential hypothesis is that the systemic inflammatory response triggered by COVID-19 infection may contribute to dysregulation in bone formation pathways, thus predisposing individuals to HO. This study aims to give a better understanding of the clinical features in patients who get HO after contracting COVID-19. It serves as a starting point for delving deeper into the potential reasons behind this connection.

## Materials and methods

**Fig. 1:**
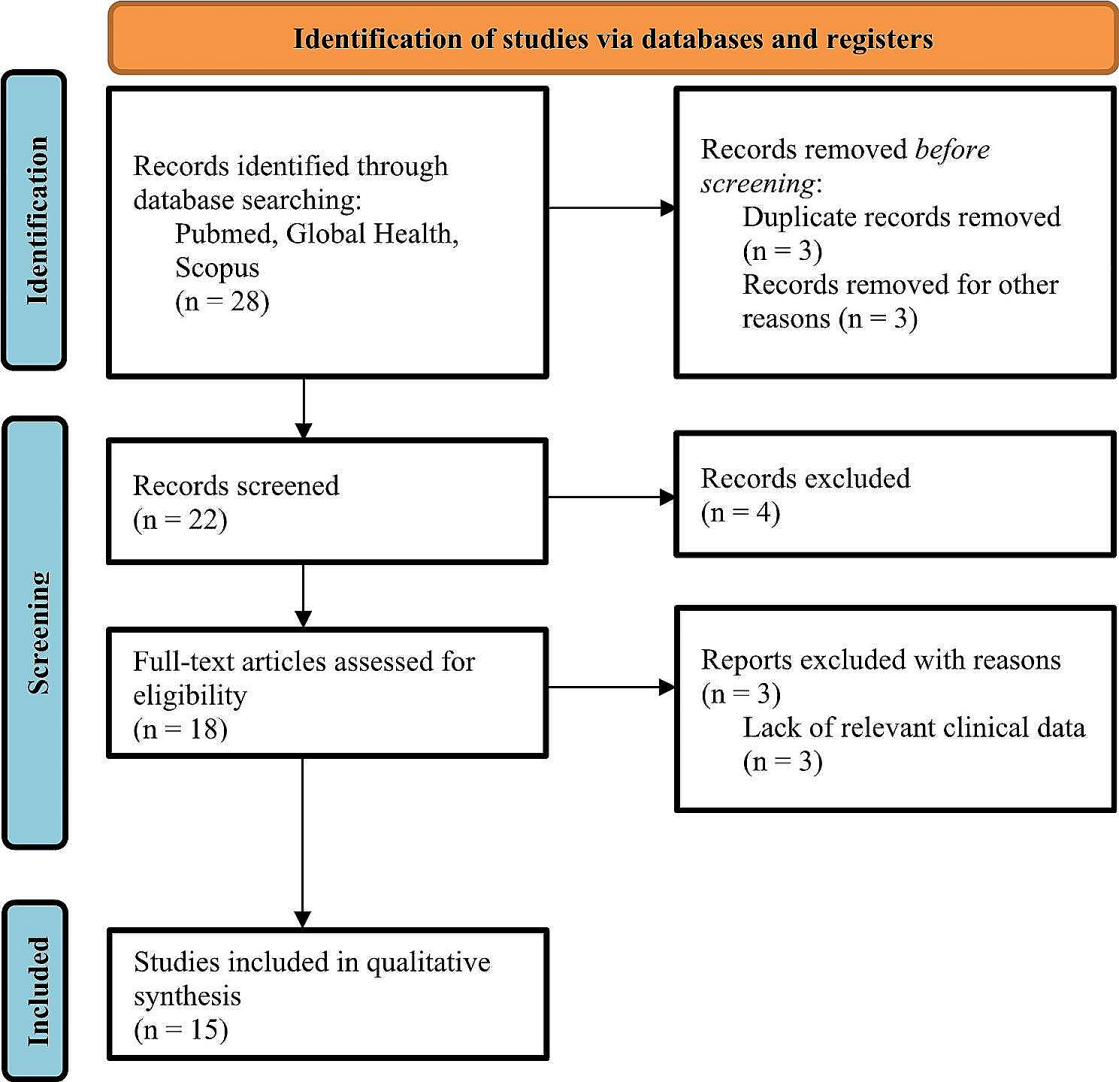
Preferred Reporting Items for Systematic Reviews and Meta-Analyses (PRISMA) flow chart

This systematic review was conducted (protocol registration: ​​*CRD42023393516*) following Preferred Reporting Items for Systematic Reviews and Meta-Analyses (PRISMA) guidelines [5], as shown in Fig. 1.

### Search strategy

We conducted a literature search for eligible articles published until January 12th, 2023, using three databases (MEDLINE/Pubmed, Global Health, and Scopus). The search strategy used the following keywords: “heterotopic ossification” or “ectopic ossification” or “myositis ossificans” and “COVID-19” or “SARS-CoV-2” or “coronavirus”. The title and abstract determined the eligibility of the case report.

### Eligibility criteria

We searched case reports and case series of HO and COVID-19. No language restriction was applied. Cases reporting COVID-19 infection and a diagnosis of HO were included. Review articles, commentaries, articles concerning fibrodysplasia ossificans progressiva, and articles with a lack of relevant clinical data were excluded.

### Study selection

Two authors reviewed independently the titles, abstracts, and full articles. These authors confirmed articles with predetermined eligibility criteria.

### Data collation and quality assessment

One author extracted data and another cross checked it. Physician collaborators helped in extracting data from articles written in other languages than English (French, Spanish, Portuguese, German, and Dutch). Subsequent details were drawn out for each case report: author, origin country, patient’s age, gender, past medical history (including other complications that the patient has developed during hospitalisation), presenting symptoms, ICU stay duration (if not specified, mechanical ventilation duration was taken into consideration), mechanical ventilation or tracheostomy use, time to HO diagnosis after COVID-19 onset (if the duration was not explicitly stated, we calculated an approximative duration in months by considering the hospitalisation duration and the time of HO symptom onset or imaging), HO diagnosis technique, HO location(s), serum alkaline phosphatase value, treatment of HO, and follow-up.

### Data analysis and synthesis

We summarised the extracted information qualitatively. Methods for synthesising qualitative data such as meta-analysis were not used, as we are providing a summary of case reports. Thus, no effect measures were calculated.

## Results

This review included 20 patients diagnosed with HO following COVID-19 infection, as reported in 15 published studies [6–20]. Table 1 provides a compilation of the extracted data from each article.

**Table 1:** Data extraction and synthesis: a summary of findings from selected articles

Author	Publication date	Country	Age(y) / Sex(M/F)	Past medical history	ICU stay (days)	Mechanical ventilation / Tracheostomy	Presenting symptoms	Time to HO diagnosis (months)	HO diagnosis technique	HO location(s)	ALP (IU/L)
Ploegmakers DJM et al. [6]	09/2020	Netherlands	59/M	Asthma, hypertension, gout, prostate carcinoma, recent right shoulder surgery	30	Mechanical ventilation	Pain and movement restrictions of the shoulders, elbows, and hips	1.5	X-rays	Bilateral shoulders	Increased
			53/M	N/A	70	Mechanical ventilation	Pain and movement restrictions in both shoulders and hips	2.5	X-rays	Bilateral hips and shoulders	397
Meyer C et al. [7]	11/2020	Belgium	64/M	Hypertension, atrial fibrillation, cervical myelopathy, sensory-motor polyneuropathy, right brachial plexus lesion	26	Mechanical ventilation	Acute pain in the groin area, painful limitation of the hip mobility in flexion, extension and rotation that limited gait and sitting	1.25	X-rays, bone scintigraphy, CT-scans	Bilateral hips	200
			73/M	Hypertension, chronic obstructive pulmonary disease, deep vein thrombosis, sensory-motor polyneuropathy	27	Mechanical ventilation	Acute pain in the groin area, painful limitation of the hip mobility in flexion, extension and rotation that limited gait and sitting	1.25	X-rays, bone scintigraphy, CT-scans	Left hip	126
			74/M	Hypertension, chronic obstructive pulmonary disease	30	Mechanical ventilation	Acute pain in the groin area, painful limitation of the hip mobility in flexion, extension and rotation that limited gait and sitting	1.25	X-rays, bone scintigraphy, CT-scans	Left hip	105
			30/M	Schizophrenia, bipolar disorder, alcohol abuse	28	Mechanical ventilation	Bilateral scapular pain with stiffness	1	X-rays, bone scintigraphy	Bilateral shoulders	200
Aziz A et al. [8]	01/2020	USA	51/F	Hypertension, type-2 diabetes, septic shock, catheter associated Enterobacter bacteraemia, Klebsiella urinary tract infection	47	Mechanical ventilation and tracheostomy	Bilateral shoulder pain and stiffness	5.5	X-rays, CT-scans	Bilateral shoulders	148
			43/F	Hypertension, septic shock	33	Mechanical ventilation and tracheostomy	Right shoulder pain	6	X-rays	Right shoulder	Normal range
Dahmen A et al. [9]	06/2021	Germany	68/M	Left humerus head fracture, bilateral pulmonary embolism, multilocular cerebral infarctions, left hemiparesis	56	Mechanical ventilation and tracheostomy	Severe pain in the left shoulder and elbow, reduced mobility in both joints	3	X-rays	Left shoulder and left elbow	299
Peters J et al. [10]	08/2021	Germany	41/M	Venous thrombosis of the right leg, occasional migrain-like symptoms	28	Mechanical ventilation and tracheostomy	Painful movement restrictions of both shoulders, pain in both hips	N/A	X-rays, bone scintigraphy	Bilateral shoulders and hips	N/A
Nieto Morales ML et al. [11]	09/2021	Spain	76/M	Pneumothorax, multiple infection processes, decubitus ulcers, generalised sarcopenia	60	Tracheostomy	Right inguinal pain with painful limitation of passive motion of the right hip, restricting the patient from sitting	4	MRI, CT-scans, bone scintigraphy	Bilateral hips	Increased
Brance ML et al. [12]	01/2021	Argentine	54/M	Deep vein thrombosis, pulmonary embolism, polyneuropathy, sacral eschar	67	Mechanical ventilation and tracheostomy	Intense pain and soft tissue swelling mainly in the hip and left shoulder affecting joint mobility	2	CT-scans	Bilateral hips and shoulders	111
da Nóbrega Danda GJ [13]	01/2022	Brazil	52/F	Hypertension, asthma, sepsis, tetraparesis secondary to a severe polyneuropathy	37	Mechanical ventilation	Pain and stiffness of the right hip	2.5	MRI	Right hip	N/A
Grosjean D et al. [14]	01/2022	Belgium	63/M	Hypertension, overweight, severe neuromyopathy	25	Mechanical ventilation	Pain and stiffness of hips and shoulders	N/A	X-rays, CT-scans, US, SPECT	Bilateral hips and right shoulder	N/A
Minjauw C et al. [15]	03/2022	Belgium	74/M	Hypertension, chronic obstructive pulmonary disease, umbilical hernia surgery, deep vein thrombosis, Pseudomonas aeruginosa pneumonia	27	Mechanical ventilation	Left hip stiffness	1.25	X-rays, CT-scans	Left hip	N/A
Van Ochten N et al. [16]	03/2022	USA	59/M	Diabetes, hypertension, pulmonary embolism on anticoagulation	43	Mechanical ventilation	Progressive hip pain	3.5	X-rays, CT-scans	Bilateral hips	N/A
Vardar S et al. [17]	03/2022	Turkey	45/M	Hypertension, mild cerebral atrophy, sepsis during ICU hospitalisation	60	Mechanical ventilation	Walking disability, stiffness, inability to stand up and move comfortably, pain in elbows and hips bilaterally	2.5	X-rays	Hips, shoulders, elbows	291
Micolich Vergara A et al. [18]	03/2022	Spain	63/M	Obesity, hypertension	134	Mechanical ventilation	N/A	5	CECT-scan	Bilateral intercostal spaces and shoulders	N/A
Liu J et al. [19]	09/2022	USA	23/F	Refractory hypoxemia, pneumomediastinum, subsegmental pulmonary embolism, disseminated intravascular coagulation, partial thickness necrosis and dry eschars of the nail and dorsal fingertips	81	Mechanical ventilation	Palpable, firm, non-fluctuant mass at the left distal medial thigh and painful limitation of active and passive motion of the left knee	N/A	X-rays, CT-scans	Left vastus medialis	N/A
Castro JM et al. [20]	10/2022	Spain	60/M	Neuropathy	60	Mechanical ventilation	Joint locking in bilateral hips, stiffness, loss of strength, bilateral paraesthesia	N/A	X-rays, CT-scans	Bilateral hips	N/A

Abbreviations: y, years; M, Male; F, Female; ICU, intensive care unit; HO, heterotopic ossification; ALP, alkaline phosphatase; IU/L, international units per litre, N/A, not applicable; CT, computed tomography; USA, United States of America; MRI, magnetic resonance imaging; US, ultrasonography; SPECT, single-photon emission computerized tomography; CECT, contrast enhanced computed tomography

The first case was reported in September 2020 [6], and the last one in October 2022 [20]. The majority of the cases were reported from Europe (13 patients, 65%), followed by the Americas (6 patients, 30%), and finally from Asia (1 patient, 5%).

The age of the patients ranged from 23 to 76 years with a median age of 59 years and a standard deviation of 14.5 years. 80% of the patients were male.

63.2% of the patients with known past medical history (*n* = 19) had hypertension, 15.8% of them had chronic obstructive pulmonary disease (COPD), and 21.1% of them had polyneuropathy of which one patient developed tetraparesis. One patient had a recent left humerus head fracture and multilocular cerebral infarction with left hemiparesis, and one patient had recent shoulder surgery. Other patients had deep vein thrombosis, pulmonary embolism, sepsis, and septic shock.

All patients required ICU care, with an average length of stay of 48.5 days (standard deviation: 26.7 days). Additionally, all patients required mechanical ventilation, and 30% underwent tracheostomy.

Of the patients for whom the presenting symptoms are known (*n* = 19), stiffness of the affected joint and pain were the predominant symptoms of HO. One patient presented soft tissue swelling [12] and one patient had a palpable mass in the knee [19]. Symptoms prompted imaging to diagnose HO in 90% of the patients, while in two cases (10%), HO was incidentally detected during imaging requested for other purposes. On average, HO was diagnosed 2.8 months after the onset of COVID-19, with a standard deviation of 1.6 months.

X-rays were the most used diagnosis technique (80%), followed by computed tomography scans (CT-scans) (60%). Bone scintigraphy was carried out in 30% of cases. 15% of the patients underwent magnetic resonance imaging (MRI) and one patient underwent a single-photon emission computed tomography (SPECT). Of the 12 cases for which serum alkaline phosphatase fluctuations were reported, 11 of them showed elevated levels.

Across all patients, HO was found to be unilocular in 30% of cases and multilocular in 70% of cases. The most common location of HO was the hip joint, accounting for 65%. Additionally, the bilateral presentation of HO in the hip was more common (69.2%) than the unilateral presentation (30.8%). Following the hip, the shoulder joint was the second most common location for HO (55%) and was also more commonly found bilaterally (72.7%) than unilaterally (27.2%).

Table 2 summarises treatments received by the patients and their follow-up. For patients with known treatment (*n* = 15), 86.7% of them received physical therapy. Three patients underwent surgery or had a surgical excision planned, and four patients were treated with nonsteroidal anti-inflammatory drugs (NSAIDs). Other therapeutic measures included corticosteroids and radiotherapy (refer to Table 3 for an overview of patient characteristics and trends). Out of 20 cases, follow-up data was available for seven patients. Among these, three experienced persistent mobility restriction, while three showed improvement in joint mobility.

**Table 2:** Patients’ treatments and follow-up

Author	Treatment	Follow-up
Ploegmakers DJM et al. [6]	Physiotherapy	Persistent pain and movement restrictions especially in the right shoulder
	Physiotherapy	Persistent movement restriction
Meyer C et al. [7]	N/A	N/A
	N/A	N/A
	N/A	N/A
	N/A	N/A
Aziz A et al. [8]	Plan for surgical excision	N/A
	Corticosteroid injection of the right glenohumeral joint, physiotherapy	N/A
Dahmen A et al. [9]	Prednisolone, physiotherapy	Reduction of pain, persistent movement restrictions
Peters J et al. [10]	NSAIDs, radiotherapy, physiotherapy	Significant pain relief and improvement of the mobility of both shoulders
Nieto Morales ML et al. [11]	Planned surgery consisting of tenotomies and muscle reconstruction, aquatic therapy, and physiotherapy in the meantime	N/A
Brance ML et al. [12]	Physiotherapy, codeine and ibandronate	N/A
da Nóbrega Danda GJ et al. [13]	Conservative treatment, rehabilitation exercises	Improvement in hip mobility and pain control
Grosjean D et al. [14]	NSAIDs for 10 days, alendronate for 2 months, pamidronate for 3 days, physiotherapy	Partial recovery with possibility of upright position and walking with a forearm walker
Minjauw C et al. [15]	Physiotherapy, NSAIDs	N/A
Van Ochten N et al. [16]	Acetaminophen and tizanidine for pain control, physiotherapy	N/A
Vardar S et al. [17]	Home-based rehabilitation program	N/A
Micolich Vergara A et al. [18]	N/A	N/A
Liu J et al. [19]	NSAIDs for pain and physical therapy	Regain of lower extremity strength and range of motion
Castro JM et al. [20]	Surgical excision	N/A

Abbreviations: N/A, not applicable; NSAIDs, non-steroidal anti-inflammatory drugs

**Table 3:** Data-driven summary of patient characteristics and trends

Characteristic	Number of patients with reported data	Results
**Age**	**20**	
Median		59 years
Standard deviation		14.5 years
**Past medical history**	**19**	
Hypertension		12 patients (63.2%)
COPD		3 patients (15.8%)
Deep vein thrombosis		4 patients (21.1%)
Pulmonary embolism		4 patients (21.1%)
Sepsis/septic shock		4 patients (21.1%)
Polyneuropathy		4 patients (21.1%)
Humeral head fracture		1 patient (5.3%)
Recent shoulder surgery		1 patient (5.3%)
Multilocular cerebral infarction with left hemiparesis		1 patient (5.3%)
**ICU admission**	**20**	20 patients (100%)
Average duration stay		48.5 days
Standard deviation		26.7 days
**Ventilation assistance**	**20**	
Mechanical ventilation		20 patients (100%)
Tracheostomy		6 patients (30%)
**Presenting symptoms**	**19**	
Joint stiffness and restriction of mobility		17 patients (89.5%)
Pain		17 patients (89.5%)
Soft tissue swelling		1 patient (5.3%)
Palpable mass		1 patient (5.3%)
**Time to diagnosis**	**16**	
Average		2.8 months
Standard deviation		1.6 months
**Diagnosis technique**	**20**	
X-rays		16 patients (80%)
CT-scans		12 patients (60%)
Bone scintigraphy		6 patients (30%)
MRI		3 patients (15%)
SPECT		1 patient (5%)
**Serum alkaline phosphatase**	**12**	
Increased		11 patients (91.7%)
**HO location(s)**	**20**	
Unilocular		6 patients (30%)
Multilocular		14 patients (70%)
Hip		13 patients (65%)
Unilateral		4 patients (30.8%)
Bilateral		9 patients (69.2%)
Shoulder		11 patients (55%)
Unilateral		3 patients (27.2%)
Bilateral		8 patients (72.7%)
Other locations		5 patients (25%)
Elbow		2 patients (10%)
Bilateral intercostal spaces		1 patient (5%)
Vastus medialis		1 patient (5%)
**Treatments**	**15**	
Physiotherapy		13 patients (86.7%)
Surgery/plan for surgical excision		3 patients (20%)
NSAIDs		4 patients (26.7%)
Corticosteroids injection		1 patient (6.7%)
Oral corticosteroids		1 patient (6.7%)
Radiotherapy		1 patient (6.7%)

Abbreviations: COPD, chronic obstructive pulmonary disease; ICU, intensive care unit; CT, computed tomography; MRI, magnetic resonance imaging; SPECT, single-photon emission computerized tomography; NSAIDs, non-steroidal anti-inflammatory drugs

## Discussion

HO is frequently divided into two groups: acquired HO, which is the most common, and rare genetic cases of fibrodysplasia ossificans progressiva and progressive osseous heteroplasia [3, 4]. Three conditions are required for HO to develop: a local environment compatible with osteogenesis, an osteogenic precursor, and a triggering event [4, 21]. Factors influencing the environment are pH, oxygen tension, micronutrients availability, and mechanical stimuli [22]. An insult triggers local inflammation with the recruitment of inflammatory cells including macrophages, lymphocytes, and mast cells, damaging skeletal muscle cells, which launches HO formation by inducing undifferentiated cell proliferation [21].

The prevalence of HO in patients with severe COVID-19 infection remains undetermined. Nevertheless, in a study by Stoira et al. [23]. which focused on a cohort of 52 COVID-19 infected patients admitted to the ICU and subjected to CT-scans, a notably high prevalence of 19.2% was observed. According to published case reports and case series, males were more commonly affected by HO, potentially due to sex-related differences that may influence predisposition [22]. 70% of the patients developed HO in multiple locations, with the hips and shoulders being the most frequently affected joints. Interestingly, these joints are also frequently affected in conditions such as traumatic brain injuries, spinal cord injuries, and burns [22].

Traumatic brain injury and spinal injury are known causes of HO formation [3, 4, 22]. Non-traumatic brain injuries were described as possible aetiologies for HO, such as vascular or anoxic brain injuries. This risk of developing HO is correlated to the severity of the brain lesions, and a higher occurrence in diffuse brain lesions was pointed out rather than focal brain lesions. It might be the result of mesenchymal cell differentiation into osteoblasts in ectopic tissues such as muscles, due to an anoxic insult [24]. Dahmen A. et al. [9] indicated that the patient had a prior history of multilocular cerebral infarction resulting in left hemiparesis, which could have contributed to the triggering of HO in addition to a humeral head fracture, with COVID-19 potentially confounding the situation.

Prolonged immobilisation and hypoxia have been identified in the literature as potential risk factors for HO [3, 22]. Since the majority of reported cases involve prolonged stays in the ICU with mechanical ventilation or tracheostomy, this is a potential confounding factor for HO development. This finding is consistent with the study conducted by Stoira et al. where HO was linked to extended periods of mechanical ventilation and prolonged hospital stays [23]. Additionally, mechanical ventilation can induce a proinflammatory state [25], which may further contribute to the development of HO.

Mesenchymal cell function is influenced by type two diabetes, which can contribute to bone emergence [21]. Two patients were reported with diabetes mellitus in their past medical history, which could exacerbate the development of HO.

SARS-CoV-2 affects mostly the higher respiratory tract but can also affect the lower respiratory tract causing pneumonia, and an ARDS in severe infections. Disease severity is not only correlated to the viral infection, but to the inflammatory response as well [2]. In severe COVID-19 infections, uncontrolled inflammation can spread and result in multi-organ damage. It implicates macrophages, monocytes, and lymphocytes generating a cytokine storm. The angiotensin-converting enzyme two (ACE2) receptor, in conjunction with the transmembrane protease, serine two (TMPRSS2) allows the entry of SARS-CoV-2 into specific cell types, in particular type two pneumocytes. While other cells, such as smooth muscle cells, synovial cells, and articular cartilage, have been found to express these proteins, the musculoskeletal system is also a potential target for the viral infection. In addition to cytokines and a proinflammatory condition, it could possibly lead to muscle and joint diseases [25]. A clinical trial is necessary in order to demonstrate the relevance of this hypothesis. Furthermore, Davis et al. reported a case in 2012 of HO after prolonged intubation due to H1N1 influenza, highlighting the potential link between HO and ARDS caused by H1N1 infection. This underscores the need for further investigation into the association between HO and infection-related ARDS, offering potential avenues for future research in understanding the underlying mechanisms and developing targeted interventions for prevention and treatment [26].

HO’s diagnosis is based on the clinical history and on radiographic imaging, which has been performed in the majority of the reported cases. The most commonly reported patient complaints were joint stiffness, restriction of mobility, and pain. Radiography and CT-scans are the gold standards for diagnosis, although three-phase bone scintigraphy is the most sensitive medical imaging to detect HO, it is also recommended for follow-up and to determine the accurate stage for surgical excision. Moreover, ultrasonography (US) is an imaging technique that is safe, affordable and easy to use. It is sensitive for detecting soft tissue lesions and calcification. Its bedside application is particularly beneficial for bed-confined patients, while also enabling quantitative assessment of HO progression during rehabilitation through variations in grey-scale values across different stages of HO maturation [4]. Serum alkaline phosphatase levels, calcium, and phosphorus are not reliable markers for diagnosis, nor for prognostication of HO [22]. We note an elevation of serum alkaline phosphatase in 91.7% of the cases. This could serve as a potential indicator of HO development. However, further confirmation is required.

Treatment for HO is divided into two categories: prophylaxis for high-risk patients, and management of already developed ectopic bone. In prophylaxis, low-dose radiation and NSAIDs tend to deliver the same result, the latter being less costly. Physical therapy is controversial in the management of formed HO but is the most commonly used treatment in the patients included in the case reports of this review. Surgical excision is recommended when the ectopic bone growth has matured and a functional deficit persists [3, 4, 22]. Although there are no specific guidelines for treating patients who have developed HO after contracting severe COVID-19 infections, they present several risk factors that predispose them to HO. Therefore, prophylaxis could be employed in such cases. Some of the treated patients had residual effects, particularly reduced mobility, which could ultimately result in a decreased quality of life. New therapy lines targeting specific mediators are being tested and are giving promising effects like targeting the hypoxia-inducible factor 1-alpha that normally stimulates endothelial cell precursors subsequently to ischemia [22], or stimulating the retinoic acid receptor (RAR) that is a chondrogenesis’ inhibitor, or inhibiting the bone morphogenic protein (BMP) pathway implicated in the differentiation of the progenitor cells to endochondral differentiation or chondrogenesis lineage [21].

It is important to acknowledge several limitations of our study. Firstly, our study primarily relies on a review of case reports, which inherently presents limitations related to data consistency and comprehensiveness. Secondly, due to the nature of our study, we lack an accurate count of these cases, which impedes our ability to calculate the prevalence of HO within the population of COVID-19 patients. Additionally, the absence of quantitative analysis, including outcome and effect measures, limits our capacity to draw definitive conclusions about the clinical impact and outcomes associated with HO in this context. Furthermore, none of the included case reports provided data on bone density, which could have shed light on the relationship between bone resorption following immobilisation and HO development. In light of these limitations, our study serves as a preliminary exploration of HO in the context of COVID-19, emphasizing the need for more extensive and rigorous research in the future to address these shortcomings and provide a more comprehensive understanding of this phenomenon.

## Conclusion

This systematic review provides a comprehensive overview of the clinical characteristics, diagnostic results, treatment options, and outcomes related to HO in COVID-19 patients. The study included 20 COVID-19 patients who developed HO. Most of them had at least two independent risk factors for developing HO, such as prolonged immobilisation and mechanical ventilation. The link between SARS-CoV-2 and HO remains uncertain, and multivariate analysis with adjustment for these risk factors are required. Although there is some evidence suggesting that SARS-CoV-2 might be targeting cells of the musculoskeletal system, it is unclear whether this is related to the development of HO. HO should nonetheless be suspected in patients with prolonged immobilisation, mechanical ventilation, and presenting joint pain and stiffness. This condition can have a significant impact on the patient’s quality of life, and its diagnosis is typically confirmed through radiographic imaging, which is considered the gold standard. The treatments of HO are controversial, and new studies are being conducted to explore new therapy lines. Therefore, it is important to continue investigating this pathology to identify effective treatment options and improve patient outcomes.

## Data Availability

All data generated or analysed during this study are included in this published article.

## Abbreviations

COVID-19: coronavirus disease 2019
SARS-CoV-2: severe acute respiratory syndrome coronavirus two
ICU: intensive care unit
HO: heterotopic ossification
COPD: chronic obstructive pulmonary disease
CT: computed tomography
MRI: magnetic resonance imaging
SPECT: single-photon emission computed tomography
NSAIDS: nonsteroidal anti-inflammatory drugs
ACE2: angiotensin-converting enzyme 2
H1N1: hemagglutinin 1 neuraminidase 1
TMPRSS2: transmembrane protease, serine 2
RAR: retinoic acid receptor
BMP: bone morphogenic protein
